# Multifunctional Biopolymer Films Based on Gelatin and Chitosan Enriched with Plant Extracts: From Functional Characterisation to Food Application and Environmental Impact

**DOI:** 10.3390/ma19102009

**Published:** 2026-05-12

**Authors:** Wiktoria Grzebieniarz, Nikola Nowak-Nazarkiewicz, Joanna Tkaczewska, Agnieszka Cholewa-Wójcik, Michał Kopeć, Krzysztof Gondek, Helena Duma, Ewelina Jamróz

**Affiliations:** 1 Department of Chemistry, University of Agriculture, Balicka 122, 30-149 Krakow, Poland; 2 Department of Animal Product Technology, Faculty of Food Technology, University of Agriculture, Balicka 122, 30-149 Krakow, Poland; 3 Department of Packaging and Logistics Processes, Cracow University of Economics, Rakowicka 27, 31-510 Krakow, Poland; 4 Department of Agricultural and Environmental Chemistry, Faculty of Agriculture and Economics, University of Agriculture, Al. Mickiewicza 21, 31-120 Krakow, Poland

**Keywords:** gelatin–chitosan films, active packaging, plant phenolic extracts, antioxidant activity, functional properties, salmon preservation

## Abstract

In the present study, innovative active gelatin–chitosan films enriched with blackberry (ACTIVE-BF) and sage flower (ACTIVE-SF) extracts were developed and comprehensively characterised with regard to their physicochemical, functional and environmental properties. The incorporation of phenolic compounds increased the film’s UV–Vis (ultraviolet–visible spectroscopy) absorbance, confirming the presence of chromophoric groups and the improvement of light-barrier properties. FTIR (Fourier Transform Infrared Spectroscopy) analysis revealed hydrogen bond formation and intermolecular interactions between polyphenols and the –OH/–NH groups of the biopolymer matrix, which enhanced the structural stability of the films. Adding blackberry and sage extracts slightly increased the hydrophilicity and solubility of the films (40–48%), without significantly affecting their water vapour transmission rate (531–547 g/m^2^·d). The obtained films exhibited strong antioxidant activity, with FRAP (Ferric Reducing Antioxidant Power) values ranging from 17.75 to 40.83 mM Trolox/mg, DPPH (2,2-diphenyl-1-picrylhydrazyl) radical scavenging capacity between 42.58 and 46.88%, and metal chelating ability up to 50.82%. During the nine-day storage of salmon fillets at 4 °C, the active films effectively inhibited microbial growth (reduction of 1.5–2.1 log CFU/g) while maintaining pH stability (6.2–6.4). Respiration activity confirmed environmental safety. The developed materials represent biodegradable, multifunctional films with high potential for application as sustainable active packaging for perishable food products.

## 1. Introduction

The modern food industry faces the challenge of reconciling growing consumer demands with the environmental issues arising from mass production and the disposal of packaging materials [1]. Plastics, which have so far served as the primary raw material in packaging production, are characterised by durability, low cost and functionality. However, their widespread use leads to the excessive accumulation of waste in the environment, contributing to the degradation of terrestrial and aquatic ecosystems [2]. According to estimates, less than 10% of global plastic production is recycled, while the remainder ends up in landfills, is incinerated or enters the natural environment, where it fragments into micro- and nanoplastics. These pollutants not only pose a threat to biodiversity but also penetrate the food chain, becoming a tangible risk to human health [3]. For this reason, the search for alternative, environmentally friendly packaging materials has become a research priority in the area of food science and polymer technology [4].

To mitigate these issues, biopolymers—natural polymers that come from renewable substances—are emerging as potential strong, sustainable competitors to synthetic materials used in packing due to their biodegradable properties. The most extensively studied examples include polysaccharides (for example, starch, cellulose or chitosan) and proteins (e.g., gelatin). Of particular importance is the ability to combine these materials into composite structures that integrate high mechanical and barrier properties with bioactivity [5].

Gelatin—a product of partial collagen hydrolysis—is widely applied as a film-forming material due to its capacity to produce transparent and flexible films having favourable oxygen barrier properties. Nonetheless, its main drawbacks include high hygroscopicity and limited mechanical resistance in conditions of elevated humidity. Chitosan, on the other hand, as a product of chitin deacetylation, is characterised by antimicrobial properties, biocompatibility and good film-forming ability. The combination of gelatin and chitosan makes it possible to produce films having better mechanical and barrier properties compared to those made from each biopolymer individually. Moreover, it allows active compounds to be incorporated, which enhance the material’s functional properties [6,7,8].

More recently, particular attention has been devoted to the enrichment of biopolymers with plant extracts, which serve as sources of natural antioxidants and compounds exhibiting antibacterial activity. This approach aligns with consumer expectations favouring a “clean label” and natural ingredients, as well as with the global trend towards reducing the inclusion of chemical additives to foods. Incorporating polyphenol-rich extracts in a biopolymer matrix not only improves film biological activity, but it also enhances their oxidative stability and resistance to UV radiation [9].

Among plant sources of bioactive compounds, blackberries (*Rubus fruticosus*), as well as the leaves and flowers of common sage (*Salvia officinalis*), hold a particularly prominent position. Blackberries are distinguished by their exceptionally high content of anthocyanins, such as cyanidin-3-glucoside and delphinidin-3-glucoside, which demonstrate strong antioxidant activity and the ability to absorb UV radiation within the 280–500 nm range [10]. Common sage (*Salvia officinalis*), in turn, is characterised by an abundance of phenolics, such as rosmarinic and carnosic acids, as well as carnosol, which demonstrate potent antioxidant properties alongside broad-spectrum antimicrobial efficacy against Gram-positive and -negative bacteria [11]. Incorporating these extracts into the biopolymer matrix can therefore considerably enhance its functionality, transforming it into an active packaging material.

The application of such solutions is particularly important regarding food products prone to spoilage, i.e., fish and seafood. Atlantic salmon (*Salmo salar*), rich in polyunsaturated fatty acids, represents a valuable component of the human diet, but it is also highly susceptible to microbial spoilage and oxidative degradation. Even after only a few days of refrigerated storage, an increase in microbial load, lipid degradation, and a decline in the sensory quality of the meat can be observed [12] ntegrating active and intelligent biopolymer-based films can effectively prolong the shelf-life of these products, while adhering to sustainable development standards [13].

In light of the presented data, the objective of the present research was to create and characterise biopolymer-based films composed of gelatin and chitosan, as well as their variants enriched with plant extracts—blackberry (ACTIVE-BF) and sage (ACTIVE-SF). An in-depth assessment regarding the physicochemical, mechanical, barrier, antioxidant and antimicrobial properties of these materials was carried out, followed by an evaluation of their applicability as active packaging for Atlantic salmon (*Salmo salar*) fillets stored in refrigerated conditions. This study is centred around the production of innovative and sustainable packaging materials that can serve as a feasible substitute for traditional synthetic materials, while meeting the expectations of modern consumers and requirements of the food sector.

## 2. Materials and Methods

### 2.1. Materials

Pork gelatin (GEL) and glycerol were supplied by Chemland (Stargard, Poland). Low molecular weight chitosan (CHIT), with a 90% deacetylation degree, was acquired from Pol-Aura (Zabrze, Poland). Sage flowers (SFs) were provided by Plantago (Działdowo, Poland), whereas blackberry fruits (BFs) were procured from HiFOOD (Kolbuszowa, Poland). Fresh salmon fillets were purchased at a local wholesale retailer (Makro Cash and Carry, Warsaw, Poland).

### 2.2. Film Preparation and Properties

#### 2.2.1. Preparing Extracts from Blackberry Fruit Extract (BF) and Sage Flowers (SFs)

In this experiment, 10 g dry matter of blackberry fruits (or sage flowers) were mixed distilled water in the volume of 100 mL. The mixture was shaken for 60 min at a temperature of 80 °C. Following incubation, the solution was subjected to one-time filtering using Whatman No. 1 filter paper in order to achieve a clear extract, with the solid residue being removed and excluded from further processing.

#### 2.2.2. Film Preparation

The polymer matrix was composed of the following two biopolymer solutions: chitosan (CHIT) and gelatin (GEL). Chitosan (1% *w*/*v*) was prepared within an aqueous solution of 2% acetic acid and subsequently blended with gelatin (7% *w*/*v*) at a ratio of 4:1. Glycerol served as the plasticizing agent for all of the polymers at a concentration of 1 mL per gram of polymer dry weight.

The biopolymer control film (BC) was formulated by incorporating distilled water in the amount of 100 mL, whereas the active films were made by adding 100 mL of the respective extract, i.e., for the ACTIVE-BF film, 100 mL of blackberry fruit extract (BF) and for the ACTIVE-SF film, 100 mL of sage flower extract (SF) was combined.

**Figure 1:**
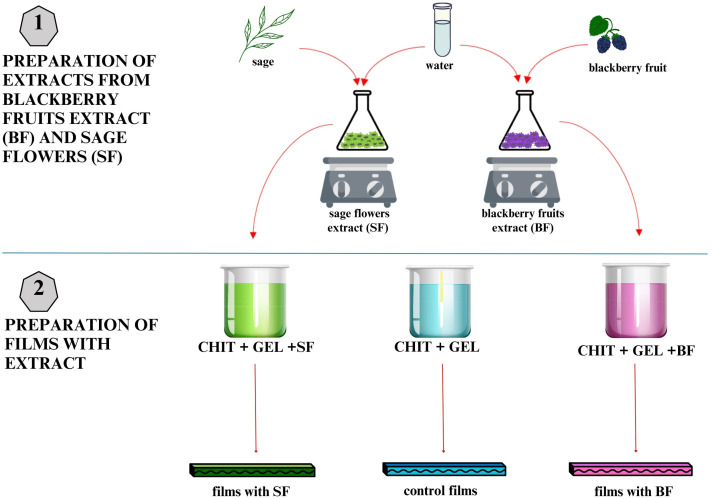
The preparation procedure of extracts and films.

The solutions that were cast in moulds were dried at ambient temperature under constant airflow. Once fully dried, the films were carefully removed from the moulds and subjected to testing. The steps involved in the preparation process are presented in Figure 1.

### 2.3. Methods

#### 2.3.1. UV–Vis Spectroscopy Analysis

The biopolymer films’ UV–Vis absorption spectra were recorded using the Shimadzu 2101 scanning spectrophotometer (Shimadzu, Kyoto, Japan) within the 300–700 nm wavelength range [14].

#### 2.3.2. FTIR Spectroscopy Analysis

The spectra were obtained via the Nicolet iS5 FTIR spectrometer (Thermo Fisher Scientific, Waltham, MA, USA) over the wavenumber range of 4000–700 cm^−1^.

#### 2.3.3. Film Thickness

The thickness of the films was determined with a precision of 1 μm using the Mitutoyo No. 7327 micrometre (Kawasaki, Japan). Measurements were taken at five different points, each located at least 10 mm from the film edge [14].

#### 2.3.4. Moisture Content and Water Solubility

Prior to evaluation, the films were equilibrated in a desiccator containing a saturated aqueous Mg(NO_3_)_2_ solution (RH ≈ 53%) in ambient conditions for a 48 h period, following the procedure described byPastor, et al. [15]. Square samples (20 mm × 20 mm) were then cut and next weighed (with a 0.0001 g accuracy) in order to determine the initial mass (W_1_). The samples were subsequently subjected to drying at a temperature of 70 °C for a period of 24 h to establish the dry weight (W_2_). To assess water solubility, each film was immersed in 30 mL of distilled water and stored at room temperature for 24 h. After incubation, the samples were blotted with filter paper and reweighed (W_3_). For each film, five independent measurements were performed [16]. Based on these values, the content of water and its water solubility were determined using the equations given below:

Water content [%] = [(W_1_ − W_2_) × 100] ÷ W_1_; (1)

Solubility [%] = [(W_1_ − W_3_) × 100] ÷ W_1_. (2)

#### 2.3.5. Water Vapour Transmission Rate (WVTR)

The analysis was carried out following standard [17] ISO 2528:2017. Silica gel was introduced into the glass vessels and sealed with biopolymer control (BC), ACTIVE-BF or ACTIVE-SF films. The vessels were then put into a controlled-climate chamber set to 25 °C and with relative humidity at 75%. The weight of the films was determined at predetermined time intervals, and the water vapour transmission rate (WVTR) was established on the basis of the increase in film weight. Prior to testing, the samples were prepared and conditioned in accordance with standard procedures. For each type of film, five replicates were analysed. The WVTR was estimated in accordance with the formula:

(3) $$WVTR=\frac{∆m}{A\times t}\times 240$$

where WVTR is the water vapour transmission rate [g/(m^2^⋅d)], Δm is the mass increase during the measurement period [mg], A is the exposed film area [cm^2^], and t is the measurement time [h]. The constant 240 is a unit conversion factor derived from: conversion of milligrams to grams (÷1000), conversion of square centimetres to square meters (×10,000) and conversion of hours to days (×24), which gives:

(4) $$240=\frac{10,000\times 24}{1000}$$

#### 2.3.6. Mechanical Properties

Mechanical properties, including maximum breaking load (MBL), tensile strength (TS), Young’s Modulus and elongation at break (EAB), were determined using the Shimadzu EZ testing machine (Kyoto, Japan) in accordance with the ASTM D882-02 standard [18].

Each analysis was performed in five replicates (*n* = 5 × 4). Before the test, the samples were subjected to temperature conditions of 23 °C and relative humidity of 50% for a duration of 24 h [16].

#### 2.3.7. Antioxidant Film Properties

##### Iron Ion Reduction Ability (FRAP Method)

The reducing power of the control film (BC) and the film enriched with the BF and SF extracts were determined using a modified version of the procedure as reported by Benzie and Strain [19]. The FRAP reagent was prepared immediately prior to each measurement and consisted of an acetate buffer (pH 3.6), 20 mM FeCl_3_ as well as 10 mM of a TPTZ (2,4,6-tripyridyl-s-triazine) solution in 40 mM HCl, which underwent mixing at a 10:1:1 (v/v/v) ratio. For sample preparation, the film in the amount of 100 mg was dissolved in 10 mL of distilled water comprising a water bath at a temperature of 50 °C, yielding an extract with a concentration of 10 mg/mL. Subsequently, a 0.4 mL volume of the sample was incubated with 3.6 mL of FRAP (Ferric Reducing Antioxidant Power) solution at 37 °C in the absence of light. This step lasted 30 min. Absorbance was then recorded at 595 nm via the Helios Gamma UV-1601 spectrophotometer (Thermo Fisher Scientific, Waltham, MA, USA). The antioxidant activity was expressed as µmol Trolox equivalents per 1 mg of film. All analyses were carried out in quintuplicate (*n* = 5 × 4) [14].

##### Free Radical Scavenging (DPPH Method)

To prepare the film extracts (10 mg/mL), 100 mg of film sample and 10 mL aliquot of distilled water were used to dissolve the 100 mg sample in a water bath set to 50 °C until complete solubilisation was achieved. An aliquot totalling 2.8 mL of the extract was then mixed with 0.2 mL of a 0.1 mM DPPH (2,2-diphenyl-1-picrylhydrazyl) solution in ethanol and maintained in the dark for 30 min. Following incubation, absorbance was recorded at 517 nm using the Helios Gamma UV-1601 spectrophotometer (Thermo Fisher Scientific, Waltham, MA, USA). A control sample, in which distilled water replaced the film extract, was used for comparison. The antioxidant activity was expressed as percentage inhibition. All measurements were carried out in quintuplicate (*n* = 5 × 4) [14].

##### Metal Chelating Ability

For the analysis, the film extract (10 mg/mL) in the amount of 1 mL was combined with 50 μL of 2 mM FeCl_2_ solution and distilled water at a volume of 1.85 mL, and next, 100 μL of 5 mM ferrozine was added. A 10 min reaction period at ambient temperature was maintained. Subsequently, absorbance was recorded at 562 nm, and the results of the analyses were given as percentage inhibition.

#### 2.3.8. Antimicrobial Property Analysis

##### Microorganisms Used in Analysis and Antimicrobial Properties

In the research, the following nine microorganisms were used: *Enterococcus faecalis*, *Candida albicans*, *Candida krusei*, *Staphylococcus aureus*, *Aspergillus brasiliensis*, *Aspergillus flavus*, *Escherichia coli*, *Pseudomonas aeruginosa* and *Salmonella enterica*. To conduct the assay, 90 mm Petri dishes were filled with 10 mL of a solidified agar medium, using the Müller Hinton Agar 2 for bacterial strains and Sabouraud Glucose Agar for yeasts. Film specimens measuring 20 × 20 mm were placed onto the surface of the agar using aseptic techniques and then preincubated at 37 °C for 24 h. Subsequently, an additional layer of molten agar inoculated with a standardised microbial suspension was poured onto the plates. Microbial growth was then assessed visually, both directly on the film surface and in the surrounding area. All determinations were carried out in triplicate [14].

#### 2.3.9. Ecotoxicity Testing

The ecotoxicity assessment was conducted in accordance with the procedure outlined by Jasińska, et al. [20]. Analysis of cress growth (*Lepidium sativum* L.) was carried out in sealed vessels using aqueous film extracts. This process was brief. The samples containing 0.258 ± 0.002 g of seeds were analysed, and the resultant CO_2_ concentration served as a measure of the toxicity degree. These analyses were performed in duplicate.

#### 2.3.10. Film Biodegradation Evaluation

Evaluation of the films’ biodegradation and respiratory activity was conducted in accordance with the protocol reported by Tkaczewska, et al. [21], applying certain modifications. To assess biodegradability degree, single-layer films were subjected to incubation following the International Organisation for Standardisation’s protocol No.1485-1:2005 [22]. The material’s respiratory activity at the time of stimulated composting was determined in controlled conditions, adhering to the method reported by Tkaczewska, et al. [21], with certain adjustments. For the incubation, 1 g (dry weight) of each film was momentarily put in 9 g of vermicompost and incubated at 45 ± 1 °C for 28 days. The incubation process of these films was performed in 2.5-dm^3^ vessels.

The respiratory activity was monitored manometrically with the use of Oxi-Top^®^ equipment (WTW Oxi-Top (Xylem) Xylem Inc. 301 Water Street SE, Suite 200 Washington, DC 20003 United States). The oxygen consumption, resulting from the inherent biological processes within the sample, was determined through recorded pressure fluctuations proportional to the oxygen uptake. Changes in pressure readings were documented at 60 min intervals. The resultant CO_2_ equivalent was absorbed by a 1 mol/dm^3^ NaOH solution placed inside the vessels. Finally, the respiratory activity was normalised to the dry weight of the materials. All analyses were conducted in triplicate.

Evaluation considering the biodegradation extent of the materials was further associated with the fractional composition of compounds that are humic. This was established with reference to the method proposed by Schnitzer (the Tiurin method) [23]. The humic compounds underwent extraction form the materials via 0.5 mol·dm^−3^ NaOH solution. Following the precipitation of humic acids (Cha) with 2 mol·dm^−3^ H_2_SO_4_, the carbon content of the fulvic acid fraction (Cfa) was verified in the remaining alkaline extract after separation via filtration. Regarding humic acids, (Cha) carbon content was subsequently derived by subtracting the Cfa value from the total carbon content of the initial extract. The Cha/Cfa ratio was established as the quotient of the carbon content in both fractions. All analyses were conducted three times [23].

#### 2.3.11. Impact of Innovative Film Packaging on Quality Retention with Regard to Shelf-Life of Refrigerated Salmon Fillets

##### Preparation of Sample

For sample preparation, salmon fillet portions of 100 g were weighed and subsequently wrapped with biopolymer film (BC) as well as with ACTIVE-BF and ACTIVE-SF films. A synthetic film was applied as the control (C). All prepared samples were placed in PET trays to ensure proper protection during storage and were then kept in refrigerated conditions at 4 °C for a nine-day period. Analyses were performed at five time intervals as follows: on days 0, 3, 6 and 9 of storage. For each measurement, three samples from each group were randomly selected, which ensured representative results and minimised the influence of biological variability of the raw material [14].

##### Microbiological Analysis of Salmon Fillets

Microbiological analyses were performed on samples that were prepared according to ISO6887-1:2017 [24]. The total viable count of the microorganisms was determined using the pour plate technique on the Plate Count Agar (PCA; Biomaxima, Warsaw, Poland). The plates were subjected to incubation at a temperature of 30 °C for a period of 48 h, and the procedure was conducted in accordance with ISO 4833-1:2013 [25]. To evaluate psychrotrophic bacteria, the same culture medium and methodology were applied; however, the incubation was extended to 240 h at 6.5 °C, following the ISO 17410:2019 standard [26].

In addition, yeast and mould (YM) populations were quantified to assess the fungal microbiota. For this purpose, the surface spread method was employed using the Dichloran Rose Bengal Chloramphenicol (DRBC) Agar (Biomaxima, Warsaw, Poland). Inoculated plates underwent incubation (25 °C, 120 h) to allow sufficient growth and differentiation of fungal colonies [14].

##### Determination of Lipid Peroxidation Using TBARS (Thiobarbituric Acid Reactive Substances) Method

At this stage of the analysis, 5 g from the sample was weighed and then mixed with 0.1% trichloroacetic acid in the amount of 25 mL (TCA; Sigma-Aldrich, St. Louis, MO, USA). The mixture was homogenised and subsequently centrifuged at 10,000× *g* for 15 min. Following centrifugation, 4 mL of the resulting supernatant was collected and transferred into a clean test tube. To this aliquot, 4 mL of 20% TCA solution comprising 0.67% thiobarbituric acid (TBA; Sigma-Aldrich, St. Louis, MO, USA) and butylated hydroxytoluene (BHT) were added. The ready mixture was incubated in a water bath (temperature: 90 °C, duration: 30 min). Continuous mixing was performed during this time. Post-incubation, the samples were cooled down to ambient temperature and also centrifuged in analogous conditions one more time (10,000× *g*, 15 min). The supernatant’s absorbance was then recorded using the Helios Gamma UV-1601 spectrophotometer (Thermo Fisher Scientific, Waltham, MA, USA) at 532 nm and adjusted to a background absorbance of 600 nm. The results of the analysis were calculated as mg TBARS/kg per product, applying the molar extinction coefficient of malondialdehyde (155 mM^−1^ cm^−1^) [14].

##### pH Analysis

The pH level of salmon fillet samples was established aided by the CP 505 pH metre fitted with the IJ-44A Elmetron electrode (Elmetron, Zabrze, Poland). Prior to measurements, calibration of the instrument was carried out using standard phosphate buffer solutions at pH 4.0, 7.0, 12.0 and temperature compensation was applied. Each sample was diluted 1:1 (*v*/*v*) with distilled water prior to analysis. Measurements were performed in quintuplicate [14].

#### 2.3.12. Statistical Analysis

The data are expressed as mean ± SD (*n* ≥ 3). Normality was verified implementing the Shapiro–Wilk test. One-way ANOVA (analysis of variance) was used for film properties, and two-way ANOVA (film type × storage time) for salmon storage tests. Tukey’s post hoc test was used to establish statistically significant differences at *p* ≤ 0.05.

## 3. Results and Discussion

### 3.1. Appearance, Thickness and Structural Characterisation of Films

The obtained gelatin–chitosan films exhibited a uniform and compact structure not having visible air bubbles and were detached without effort from the casting mould after drying. The control film (BC) showed a semi-transparent, yellowish colour typical of protein–polysaccharide matrices. The incorporation of plant extracts caused a noticeable colour change—the film containing blackberry extract (ACTIVE-BF) developed a pinkish-violet hue, while the film with sage extract (ACTIVE-SF) exhibited a light greenish-brown tint. These colour variations are a consequence of the anthocyanin and phenolic acid presence in the respective extracts. The film thickness was within the range of 0.14 and 0.15 mm for all samples, with no significant differences noted between the films. Thickness of the film is a key factor influencing functional properties, i.e., water vapour permeability or mechanical strength, transparency and film efficacy as active barriers protecting the packaged product from environmental factors.

The UV–Vis analysis of the obtained films allows for the evaluation of their ability to absorb UV–Vis radiation, which is crucial in the development of food packaging materials. Insufficient protection against UV radiation may accelerate photo-oxidative processes, leading to the damage of both the packaging medium and its contents [27].

**Figure 2:**
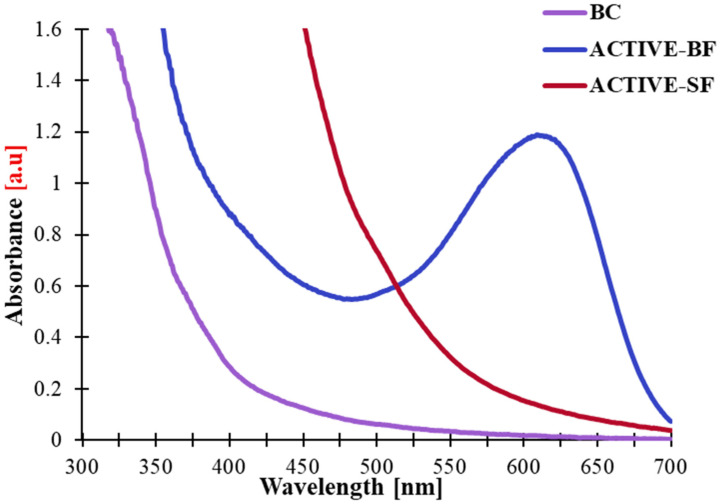
UV–Vis spectra of the designed films ranging between 300 and 700 nm.

The UV–Vis spectra of the developed films (Figure 2) revealed clear differences with regard to the control sample (BC) and the films enriched with plant extracts (ACTIVE-BF, ACTIVE-SF). The control film (BC) exhibited low absorbance within the UV region (<350 nm), indicating the absence of chromophoric compounds in the biopolymer matrix. This result is typical for polysaccharide–protein matrices, which lack aromatic chromophores and therefore show limited ability to protect against UV–Vis radiation. Consequently, such films are increasingly prone to degradation during storage [28]. The addition of plant extracts notably altered the optical characteristics of the studied substances. In the case of the ACTIVE-BF film, absorption was observed both in UV and visible regions, with a characteristic maximum at approximately 600–620 nm, typical of anthocyanins present in blackberry extract. The increase in absorbance within the visible range confirms the effective incorporation of phenolic compounds into the polymer matrix and correlates with the improvement of light barrier properties, which aligns with the findings from earlier research on gelatin- and chitosan-based films enriched with anthocyanins [29]. The ACTIVE-SF film exhibited absorption within the range of 300–450 nm, resulting from the occurrence of phenolic compounds specifically, rosmarinic and caffeic acids, as well as flavonoids including luteolin and apigenin, which are commonly found in sage flowers and known for their UV-protective properties [30].

**Figure 3:**
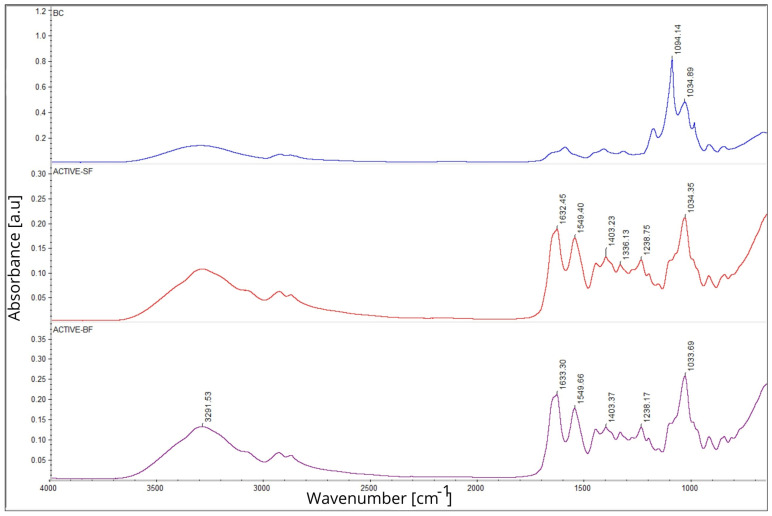
FTIR spectra of gelatin–chitosan films without extracts (BC) and those containing blackberry (ACTIVE-BF) or sage (ACTIVE-SF) extracts.

The FTIR spectra of the analysed films (Figure 3) allowed identification of characteristic functional groups present in the gelatin–chitosan matrix and detection of structural changes resulting from the incorporation of plant extracts. The differences observed between the obtained spectra clearly indicate the occurrence of chemical interactions between the polymer chains and the phenolic constituents of the extracts [31].

In all samples, a broad band within the range of 3000–3700 cm^−1^ was observed, which is associated with the vibration stretching of hydroxyl (–OH) and amide (–NH) groups. These are characteristic of gelatin and chitosan. In the films containing plant extracts, these bands became broader and more intense, suggesting that further hydrogen bonds have formed between the hydroxyl and amino groups of the polymer matrix and the polyphenols rich in –OH groups. This effect confirms enhanced intermolecular interactions and improved compatibility between the biopolymer phase and the incorporated plant extracts [31].

Between the range of 2800 and 3000 cm^−1^, C–H stretching bands characteristic of aliphatic groups were observed, being more pronounced particularly in the ACTIVE-SF sample, which may be ascribed to the presence of terpenoids and lipid compounds, for instance, carnosic acid and carnosol naturally occurring in this extract. Within the 1500–1700 cm^−1^ region, amide I (C=O, approx. 1640 cm^−1^, N–H, approx. 1540 cm^−1^) bands were detected, showing slight shifts and variations in intensity upon the incorporation of plant extracts. These changes proved that interactions occur between the carbonyl and amino groups of the gelatin–chitosan matrix and phenolic compounds, which may lead to hydrogen bond formation and alterations in the polymer structure. In the lower frequency region (1450–1000 cm^−1^), bands connected with C–O and C–N stretching vibrations were observed. The modifications in this area indicate effective integration of plant extracts within the biopolymer matrix. The observed spectral changes confirm the successful incorporation of bioactive compounds into the gelatin–chitosan structure, likely contributing to enhanced structural stability as well as enhanced functional attributes of the developed films [31].

### 3.2. Water Content and Solubility

Water-related properties are significant parameters determining the functionality of biopolymer materials intended for packaging applications, as they affect both the physical stability of the materials and the shelf-life of the stored food [32]. The results obtained for the developed gelatin–chitosan films are presented in Table 1.

The water content of the samples submitted to testing were within the range of 13.22 and 13.50%. The results showed no significant differences between the control film and those containing plant extracts. This indicates that adding blackberry and sage extracts had no significant effect on the hygroscopic equilibrium of the biopolymer matrix. A similar water content level (12–15%) for biopolymer materials based on gelatin–chitosan systems was observed by Nowak, et al. [33], who indicated that such values are typical of biopolymer films and are attributable to the occurrence of a large number of hydrophilic groups in the matrix structure (–OH, –NH_2_). Rodrigues, et al. [31] also reported that the establishment of hydrogen bonding between gelatin, chitosan and polyphenols reduces moisture fluctuations within the film structure, thereby contributing to its physical stability. The obtained results clearly suggest that the developed materials exhibited appropriate structural integrity and water-retention capacity, which are desirable features for packaging applications [31].

The water solubility values of the tested films were between 40.87 and 48.09%, with both active films (ACTIVE-BF and ACTIVE-SF) showing significantly higher values than the control sample. The increase in solubility may potentially be attributed to the existence of hydrophilic phenolic groups (–OH) in the extracts, which increase the polarity of the system and reduce the number of intermolecular interactions within the matrix. Kurek, et al. [34] demonstrated that incorporating anthocyanins and phenolic compounds into biopolymer films increases their water solubility by disrupting the polymer structure. The solubility values obtained in the present study (approx. 32–46%) fall within the typical range reported for gelatin–chitosan matrices. Similar values (50.45–56.28%) were given by Mohammadi, et al. [35] for gelatin–chitosan composite films having different biopolymer ratios, confirming that the solubility of the investigated materials is consistent with the characteristics of this type of polymer system.

From the perspective of active packaging, the observed stable water content may favour the maintenance of the material’s dimensional and mechanical stability during storage, while the increased solubility may facilitate contact between the matrix and moisture or food exudate, thereby supporting the migration (release) of phenolic fractions with antioxidant activity during product storage. However, excessive solubility may restrict its use in packaging systems requiring high moisture resistance [36].

### 3.3. Water Vapour Transmission Rate (WVTR)

The WVTR parameter is an important indicator of material suitability for packaging applications, as it reflects the ability to limit water vapour transmission between the product and the external settings [37].

The WVTR values obtained for the tested biopolymer films ranged from 531.37 to 546.67 g/m^2^·d (Table 1), with no statistical differences in statistical significance noted among the samples. The slight reduction in this parameter noted for the ACTIVE-BF film may be related to partial sealing of the matrix structure by anthocyanin compounds, which may fill microvoids between polymer chains [38]. With regard to the ACTIVE-SF film, the presence of terpenoids and phenolic acids, including carnosic acid and carnosol, may have balanced the hydrophilic effects, maintaining water vapour permeability at a level similar to that of the control film. Comparable WVTR values have been reported for chitosan-based films by Wiles, et al. [39], who demonstrated that WVTR strongly depends on the water vapour pressure gradient and at 25 °C, it may range from a few to more than 1000 g/m^2^·d. Therefore, the values obtained in the present study (~531–547 g/m^2^·d) fall within the range reported for chitosan films in conditions of higher relative humidity differences.

These results may be considered favourable from the perspective of active packaging applications, since maintaining low or stable water vapour permeability is essential for quality preservation and extending the shelf-life of stored food products [40].

### 3.4. Mechanical Properties of the Films

A biopolymer–protein film matrix should exhibit appropriate mechanical properties to ensure resistance to the typical stresses faced during transport, storage and use. The use of aqueous extracts may affect the film structure depending on their degree of dispersion and their interactions within the gelatin–chitosan network [41]. In systems based on gelatin and chitosan, the most important mechanical parameters comprise tensile strength and elongation at break, as well as Young’s modulus. They reflect the material’s resistance to rupture, its flexibility and its structural stiffness, respectively [42]. In the literature, it is also indicated that protein-based materials generally display more favourable mechanical properties than systems based solely on polysaccharides, mainly because of the presence of strong intermolecular interactions within the protein matrix [43,44].

The mechanical properties of the investigated films can be found in Table 2. The control film (BC) demonstrated the highest tensile strength (4.51 kN/m) and Young’s modulus (1016.35 N/mm^2^), indicating high stiffness and a compact structure of the biopolymer matrix. The addition of blackberry and sage extracts caused a slight decrease in these values, while in the case of tensile strength, the differences were of no statistical relevance. The mentioned effect may be attributed to a partial disruption of the hydrogen-bonding network by phenolic components of the extracts, which can compete for interactions with the carbonyl and amino groups of the biopolymer [45]. Similar relationships have been reported for films containing plant-derived additives and anthocyanins, where modifications in the hydrogen-bonding system affected the mechanical parameters of the material [29].

Significant differences were observed for elongation at break (EAB). The ACTIVE-BF film exhibited an EAB value more than twice as high (16.70%) as that of the control film (6.13%), indicating increased flexibility and greater deformation capacity. This increase may be attributed to anthocyanins present in the blackberry extract, which can act as natural plasticisers by lowering the amount of hydrogen bonds and increasing the mobility of polymer chains. This interpretation is consistent with that given in studies on gelatin–chitosan films enriched with anthocyanins, where a rise in elongation at break was accompanied by a reduction in tensile strength and elastic modulus. Qin, Wang, Tang, Chen, Wang, Cheng, Chi and Soteyome [29] demonstrated that incorporating anthocyanins into a chitosan–gelatin film induced an increase in EAB and a reduction in TS (Tensile strength), while FTIR analysis confirmed the involvement of hydrogen bonding in these changes. Similar relationships have also been summarised in review papers devoted to chitosan–anthocyanin films and anthocyanin-based intelligent packaging [46].

The analysis of Young’s modulus supports this interpretation. The marked decrease in its value for the ACTIVE-BF film (646.93 N/mm^2^) implies reduced stiffness of the system and increased susceptibility of the material to deformation. This means that the blackberry extract did not significantly impair the film strength, but rather enhanced its flexibility, which may be advantageous from the perspective of packaging applications. In active biopolymer films, such an effect is often desirable, as greater flexibility facilitates handling and reduces the risk of material cracking during storage [47].

With reference to the ACTIVE-SF film, the EAB value was comparable to the date obtained for the control sample, while the decrease in Young’s modulus was less pronounced than in ACTIVE-BF, which may indicate that the sage extract exerted a weaker plasticising effect. This is most likely related to the different phytochemical profile of the extract, particularly the presence of more hydrophobic terpenoid and essential-oil components, with their influence on film structure depending on them being compatible with the matrix and degree of dispersion within the system. In the literature on bioactive films containing essential oils and terpenoid compounds, it is indicated that such additives may modify mechanical properties in a less predictable manner than polyphenolic compounds; depending on their composition, they may act either as plasticisers or as stiffening agents, while the final effect depends on phase distribution and the nature of interactions with the polymer [48,49,50].

The achieved results confirm the incorporation of plant extracts modified the films’ mechanical properties in a controlled manner, increasing their flexibility without significantly impairing their strength. The most favourable effect was observed for the film containing blackberry extract, suggesting that anthocyanins may act as natural modifiers for the mechanical properties of biopolymer packaging materials. Similar conclusions regarding the incorporation of phenolic extracts into films were provided by Jamróz, et al. [51], who noted a deterioration in certain mechanical parameters accompanied by improved flexibility in double-layer biopolymer films.

### 3.5. Antioxidant Properties of the Films

Antioxidant properties are greatly important in the context of active films intended for application in food packaging systems, as they may limit lipid oxidation, slow down undesirable quality changes, and help preserve the sensory attributes in addition to the nutritional quality of food items. In the literature, it is emphasised that active packaging systems containing natural antioxidants, particularly plant-derived phenolic compounds, can effectively inhibit oxidative processes responsible for fat rancidity and overall quality deterioration of food [52,53]. The films’ antioxidant activity was determined via FRAP (Ferric Reducing Antioxidant Power method), DPPH (2,2-diphenyl-1-picrylhydrazyl radical scavenging method)as well as and metal ion-chelating activity assays, with the findings summarised in Table 3.

The control film (BC) exhibited very low antioxidant activity (FRAP: 0.57 mM Trolox/mg, DPPH: 12.76%, MCA: 0.00%), confirming that the gelatin–chitosan matrix itself has only a limited ability to neutralise free radicals. Incorporating the plant extracts led to a marked rise in antioxidant activity. For the film containing blackberry extract (ACTIVE-BF), the FRAP and DPPH values reached 17.75 mM Trolox/mg and 42.58%, respectively, whereas for the sage-containing film (ACTIVE-SF), they amounted to 40.83 mM Trolox/mg and 46.88%, respectively. These results clearly indicate that the bioactive compounds contained within the plant extracts were primarily responsible for the substantial improvement in the antioxidant potential of the films, which is consistent with reports on other active biopolymer films enriched with phenolic compounds [54,55].

The highest antioxidant activity was observed for the ACTIVE-SF film. This effect is likely due to the occurrence of phenolic and diterpenoid compounds characteristic of sage, particularly rosmarinic and carnosic acids, as well as carnosol, which are among the best-described natural antioxidants occurring in plants of the Lamiaceae family [56]. These compounds are capable of scavenging free radicals, donating hydrogen atoms or electrons, and interrupting radical chain reactions in lipid systems, which explains the high FRAP and DPPH values obtained for this film. In reports concerning sage extracts and related plants, it is also indicated that the presence of these constituents is responsible for the strong antioxidant properties observed both in the extracts themselves and in functional materials containing such additives [57].

In turn, the rise in antioxidant activity seen in the ACTIVE-BF films may be attributed to the presence of anthocyanins, mainly cyanidin derivatives, which—owing to their numerous hydroxyl groups—are able to form relatively stable phenoxyl radicals and thereby neutralise free radicals. In reviews regarding anthocyanins’ antioxidant activity of anthocyanins, and also studies devoted to blackberry extracts, it is implied that these compounds exhibit strong reducing power and radical-scavenging capacity, which is consistent with the increased FRAP and DPPH values recorded for the ACTIVE-BF film [58].

Metal ion-chelating activity, which was not observed in the control sample, increased to 35.97% for ACTIVE-BF and 50.82% for ACTIVE-SF. This effect confirms that the phenolic compounds present in the extracts were able to effectively bind transition metal ions, thereby limiting their catalytic involvement in oxidative reactions [59].

Therefore, the obtained results confirm including plant extracts in the gelatin–chitosan matrix effectively imparted antioxidant properties to the films. Both extracts enhanced the activity of the investigated materials; however, a stronger effect was observed for the sage extract, which may stem from the existence of greatly active diterpenoid and phenolic antioxidants. Blackberry extract also notably enhanced the films’ antioxidant parameters, indicating the considerable potential of anthocyanins as natural functional additives for biodegradable packaging materials. This conclusion is consistent with that reached in previous research on biopolymer films enriched with plant extracts, noting a clear increase in DPPH and FRAP activity following the incorporation of bioactive phenolic compounds [54,60].

### 3.6. Antimicrobial Property Analysis of the Films

Antimicrobial activity is one of the key criteria used to assess the functionality of bioactive films intended for application in active food packaging. In the literature, it is emphasised that the effectiveness of such materials depends not only on the presence of an active compound, but also on its type, concentration, ability to be released from the matrix and susceptibility of the target microorganisms [61,62].

In the experiments carried out for the gelatin–chitosan matrix and its modifications with blackberry and sage extracts, the tested bacterial and fungal strains exhibited no susceptibility to the applied treatments. All samples, including both the control film (BC) and the active films (ACTIVE-BF and ACTIVE-SF), showed no activity against *Escherichia coli*, *Salmonella enterica*, *Pseudomonas aeruginosa*, *Aspergillus flavus*, *Aspergillus brasiliensis*, *Staphylococcus aureus*, *Candida krusei*, *Candida albicans* or *Enterococcus faecalis*. Such a result does not exclude the presence of bioactive compounds in the films, but rather suggests that in the applied conditions, their antimicrobial activity was insufficient to produce a measurable inhibitory effect in the test system [63].

The lack of antimicrobial activity may be attributed to several physicochemical and biological factors. First, the concentration of plant extracts incorporated into the matrix may have been too low to reach the level of microbial inhibition observed for the pure extracts [63]. Many phenolic compounds exhibit bacteriostatic activity only above a certain threshold concentration, and their immobilisation within the film structure may limit the diffusion of active substances into the culture medium [62].

Hydrogen bonding and electrostatic interactions between phenolic compounds as well as the chitosan and gelatin chains may also lead to a partial loss of the biological activity of these compounds by reducing their bioavailability. This phenomenon is well-described for biopolymer–polyphenol systems, in which improved physicochemical properties of the material do not always correspond to a proportional increase in biological activity [64].

Both chitosan and gelatin exhibit moderate or limited antibacterial activity in neutral pH conditions, which may have further weakened any potential synergistic effect with the incorporated extracts [65]. The lack of inhibitory action towards Gram-negative bacteria, specifically *E. coli* and *P. aeruginosa*, is also consistent with literature reports indicating that the outer membrane of these microorganisms functions as a highly efficient barrier towards the majority of phenolic compounds [66].

Despite the lack of antimicrobial activity demonstrated for the in vitro tests, the occurrence of a beneficial effect in the case of in vivo conditions cannot be excluded. A similar relationship was reported by Grzebieniarz et al. [14], who noted that despite a negative result obtained for in vitro assays, the in vivo analysis revealed a positive protective effect. This phenomenon could be attributed to the progressive release of active compounds from the film into the product environment, which, in real storage conditions, may have a more effective impact on the microflora than that observed in model laboratory tests [14].

### 3.7. Ecotoxicity Testing and Biodegradation Assessment of Films

The environmental assessment of the developed films included both the ecotoxicity of the materials and their susceptibility to biodegradation in controlled composting conditions. This approach is consistent with current trends in the evaluation of biopolymer-based packaging materials, for which, in addition to technological functionality, their biological impact and behaviour after end use are also taken into account.

The results presented in Table 4 indicate that the films exhibited a similar pattern of respiratory activity. During the initial stage of incubation, covering the first 48 h, a clearly higher rate of oxygen consumption was observed, indicating intensive microbial activity towards the more readily available components of the material.

The final respiratory activity after 28 days of composting reached 177.2 ± 12.2 mg O_2_/g for BC, 167.42 ± 10.6 mg O_2_/g for ACTIVE-BF, and 166.6 ± 9.9 mg O_2_/g for ACTIVE-SF. The absence of statistically significant differences between the tested samples indicates that adding the blackberry and sage extracts had no significant effect on the final biodegradation susceptibility of the films. This means that modification of the biopolymer matrix with active compounds did not inhibit the mineralisation processes of the material in composting conditions. This is consistent with the results of earlier studies on active biopolymer films containing plant-derived additives, in which biodegradability was retained despite enrichment with extracts. In the study by Tkaczewska, et al. [67] on an active double-layer edible film, nearly complete biodegradation within a short composting period and no toxicity toward garden cress were reported. In more recent research carried out by Jasińska, et al. [20], the biodegradability of films containing plant extracts was confirmed.

The biodegradation of organic substances may proceed at varying rates, contingent upon process conditions (including biomass fragmentation and homogenisation, oxygen availability and temperature) as well as the type of additives introduced into the matrix. During this process, changes in the degree of polymerisation regarding organic substances are observed, leading to the formation of more complex humic acid structures at the expense of simpler fulvic acids, which is characteristic of progressing humification processes [68,69].

Analysis concerning the humic compounds’ fractional composition (Table 4) confirmed a favourable course of humification processes in all experimental variants, although clear differences were observed depending on the applied additive. The lowest content of extractable carbon (70.75 g·kg^−1^ d.m.) was found in the control variant (BC), while significantly higher values were obtained in the variants with additives, ACTIVE-BF (74.78 g·kg^−1^ d.m.) and ACTIVE-SF (77.25 g·kg^−1^ d.m.). A comparable tendency was exhibited by humic acids’ carbon. The value of this parameter was lowest in the BC variant (32.51 g·kg^−1^ d.m.), whereas in the ACTIVE-BF and ACTIVE-SF treatments, it increased significantly, reaching 37.56 g·kg^−1^ d.m. and 38.81 g·kg^−1^ d.m., respectively. This indicates an intensification of humification processes and the formation of more complex organic structures. This phenomenon is consistent with the literature, which suggests that organic or bioactive additives may accelerate condensation and polymerisation processes, leading to an increased share of stable humic fractions [70].

The content of fulvic acids’ carbon did not show a clear increasing trend—the lowest value was recorded for ACTIVE-BF (37.22 g·kg^−1^ d.m.), while the BC and ACTIVE-SF variants exhibited similar, higher values (38.24 and 38.44 g·kg^−1^ d.m., respectively). This may indicate their partial transformation into more complex humic fractions, which is a typical phenomenon in advanced stages of humification, during which simpler compounds undergo condensation into structures with higher molecular weight [71].

A significant indicator of the degree of humification is the Cha/Cfa ratio. A significant increase was demonstrated in this ratio in the variants with ACTIVE-BF and ACTIVE-SF additives compared to the control variant. This reflects a shift in equilibrium towards more condensed and stable forms of organic matter, characteristic of humic acids. Values exceeding one are commonly considered an indicator of an advanced humification stage and greater stability of organic matter.

The application of ACTIVE-BF and ACTIVE-SF additives enhances the humification process of organic matter, leading to an increased proportion of stable humic acid fractions and a higher degree of maturity and stability of organic matter compared to the control variant.

### 3.8. Active Properties of the Films—Research Conducted In Vivo

The refrigerated storage of fish represents a major technological challenge, as this raw material belongs to the group of highly perishable foods. This is due to its very high water activity, typically between 0.98 and 0.99, and the presence of nitrogenous compounds, free amino acids and lipids susceptible to oxidative changes. Such a combination promotes both the rapid growth of spoilage microflora and the occurrence of unfavourable chemical and enzymatic changes during storage. For this reason, one of the important trajectories for the advancement of modern packaging materials is the design of biodegradable bioactive films which, in addition to serving as barriers against gases and moisture, may also limit oxidative processes and help sustain the microbiological quality of fish products [72,73,74].

**Figure 4:**
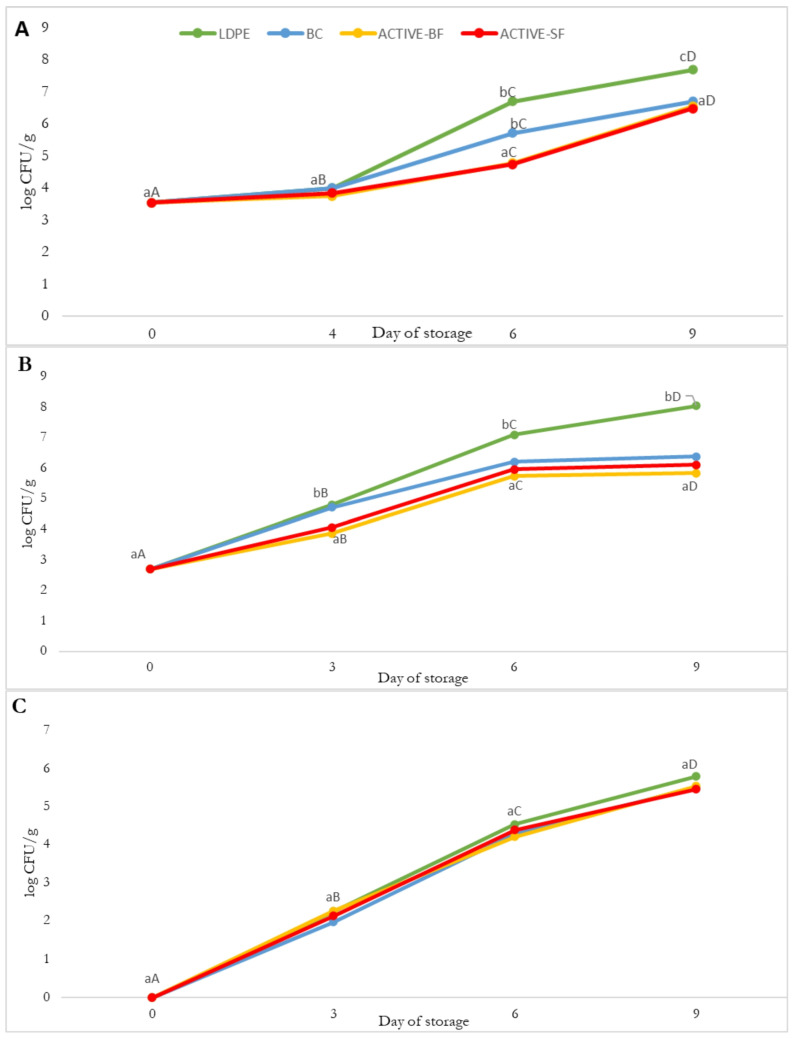
Salmon fillet microbiological contamination during nine-day storage period at temperature of 4 °C. (**A**) total aerobic bacteria; (**B**) yeasts and moulds; (**C**) total psychrotrophic bacteria. Lowercase letters—difference between groups within same day. Uppercase letters—difference within same group during storage. BC—salmon flakes, edible film coating, no extract; ACTIVE SF—salmon flakes, edible film coating, SF extract addition; ACTIVE BF—salmon flakes, edible film coating, BF extract addition; LDPE—salmon flakes, LDPE coating.

In the present study, the effect was analysed of gelatin–chitosan coatings (BC)—as well as their modifications with blackberry fruit (ACTIVE-BF) and sage flower (ACTIVE-SF) extracts—on the microbiological quality of salmon (*Salmo salar*) fillets stored for a nine-day period at a temperature of 4 °C. Changes in the total viable count (A), yeasts and moulds (B), and psychrotrophic bacteria (C) are presented in the graphs (Figure 4).

Fresh fish constitute an excellent model for studying oxidative changes occurring in food under the influence of environmental factors. They contain considerable amounts of fat and polyunsaturated fatty acids, which exhibit high susceptibility to oxidative processes. In fish lipids, up to 40% may consist of long-chain polyunsaturated fatty acids (LC-PUFAs), especially eicosapentaenoic acid (EPA) and docosahexaenoic acid (DHA), which are highly reactive towards oxygen-free radicals. Lipid oxidation causes the formation of aldehydes, ketones and other secondary products that impair the taste, odour and colour of the product, while also shortening its shelf-life [75].

#### 3.8.1. Effects of Films on Salmon Fillet Microbiological Quality in Storage Conditions

A systematic rise in the number of microorganisms was noted in all groups throughout storage, which is typical of refrigerated fish products characterised by high water activity and high availability of nutrients. Fish are among the raw materials most susceptible to spoilage, and even in chilled conditions, the growth of psychrotrophic, aerobic and fungal microflora gradually progresses over time. In the literature on coatings and films used for fish products, it is indicated that bioactive materials usually do not completely inhibit microbial growth, but rather slow down microbial proliferation through barrier effects, reduction in oxidative changes and the partial release of active compounds [34].

#### 3.8.2. Total Viable Count (TVC)

A rise in the total viable count was reported in all groups during storage; however, the dynamics of this process depended on the type of film used. Samples packaged in LDPE (low-density polyethylene) exhibited the highest TVC values, reaching approximately 8 log CFU/g after nine days, whereas in samples having the control BC film coating, the bacterial count was approx. 7 log CFU/g. The lowest values were exhibited for samples covered with active films, particularly the film containing blackberry extract (ACTIVE-BF), in which the bacterial count did not surpass 6.8 log CFU/g after nine days. These differences suggest that both the presence of phenolic compounds and the biopolymer matrix itself may have delayed the development of aerobic microflora, especially during the initial stages of storage. Comparable findings have been documented in previous research on active films used for salmon fillet storage, where samples packaged in biopolymer-based materials showed lower microbial counts than those packed in synthetic materials, with the strongest effect observed during the first days of storage [21,33,34].

#### 3.8.3. Yeasts and Moulds

In the case of fungal microflora, clear differences were observed among the samples. Those packaged in LDPE showed a rapid increase in the number of yeasts and moulds, reaching values above 8 log CFU/g after nine days. For samples packaged in BC films, the counts were approximately 1.5 log lower, whereas the lowest values were observed for the ACTIVE-SF film, in which the growth of fungal microflora was reduced by approximately 2–3 log CFU/g compared to LDPE. This effect may be due to the occurrence of phenolic acids and diterpenes in the sage extract, particularly rosmarinic acid, carnosol and carnosic acids. These compounds may disrupt the integrity of microbial cell membranes and inhibit aerobic respiration processes, thereby slowing down microbial metabolism and the division of cells [48,76,77].

#### 3.8.4. Psychrotrophic Bacteria

Psychrotrophic bacteria, mainly those belonging to the genus Pseudomonas, are among the principal causes of fish spoilage during low-temperature storage [78,79]. Their counts increased in all samples, reaching values ranging between 5.5 and 6.5 log CFU/g after nine days. However, both ACTIVE-BF and ACTIVE-SF showed a slower growth rate compared with LDPE, particularly during the first half of the storage period. The slower proliferation of psychrotrophs in the ACTIVE-BF and ACTIVE-SF samples suggests that the active films modified the microenvironment at the product surface, most likely by limiting oxidative processes and through the metal-chelating properties of phenolic compounds towards transition metal ions [66,76,80].

Although no antimicrobial activity was observed against the model strains for the in vitro tests, a clear slowdown in microflora development was noted during salmon storage. The lack of full agreement between in vitro and in vivo test results suggests that the mechanism of action of the bioactive films may not rely on a direct bactericidal effect, but rather on the modification of environmental conditions at the film–product interface, including reduced gas and moisture exchange, and also the graded release of active compounds from the matrix [81,82,83]. The results indicate that utilising active films caused an extension in the salmon fillets’ microbiological shelf-life by approximately one to three days compared to LDPE. The strongest protective effect was observed for yeasts and moulds with respect to ACTIVE-SF, whereas the blackberry extract (ACTIVE-BF) showed a more favourable effect against aerobic bacteria during the initial stage of storage.

#### 3.8.5. Effects of Films on pH and TBARS of Salmon Fillets During Storage in Refrigerated Conditions

In the current research, the effect was investigated of active gelatin–chitosan films containing blackberry fruit (ACTIVE-BF) and sage flower (ACTIVE-SF) extracts on lipid oxidation and pH changes in salmon fillets subjected to a nine-day storage period at 4 °C (Table 5). Fish products, especially those rich in long-chain polyunsaturated fatty acids, belong to the group of raw materials that are particularly susceptible to lipid oxidation [34].

The value of TBARS for the fresh fillets was 0.30 mg MDA/kg (malondialdehyde per kilogram), confirming the high freshness of the raw material at the beginning of the experiment. At subsequent stages of storage, an increase in thiobarbituric acid reactive substances was observed, indicating progressive lipid peroxidation. The lowest values for TBARS (thiobarbituric acid reactive substances) noted during the period of storage were generally recorded for samples packaged in the LDPE film (0.18–0.30 mg MDA/kg). With reference to the active films, the pattern of variations was more complex: in ACTIVE-BF samples, higher TBARS values were already observed on days three and six, whereas in ACTIVE-SF samples the highest value in the entire experiment was recorded after nine days, reaching 1.37 mg MDA/kg. The achieved results suggest that, regarding the materials under study, LDPE provided the most effective protection against lipid oxidation, which could be ascribed to its excellent oxygen barrier properties. This is consistent with literature data suggesting that the lipid oxidation rate in fish products is dependent on not only the presence of antioxidant compounds, but also on the oxygen and water vapour permeability of the packaging materials [34,41].

According to the widely accepted classification of oxidative quality in fish, TBARS values below 0.58 mg MDA/kg correspond to a fresh product, values within the range of 0.58–1.51 mg MDA/kg indicate a somewhat rancid but nonetheless still satisfactory product, whereas values exceeding 1.51 mg MDA/kg are characteristic of spoiled fish [84]. On this basis, it can be concluded that all tested samples remained within the acceptable quality range after nine days of storage, although the highest degree of oxidative deterioration was observed in the ACTIVE-SF samples.

The initial pH of fresh fillets was 6.34 ± 0.06. During storage, changes in this parameter varied depending on the type of film applied. On days 3 and 6, the lowest values for pH values were noted in the ACTIVE-BF samples (6.29 ± 0.01 and 6.19 ± 0.03, respectively), whereas the highest values were observed for samples packaged in LDPE and BC. Following the nine-day storage period, the highest pH was still found in the LDPE samples (6.68 ± 0.10), while the active films maintained lower and more stable values (6.35 ± 0.14 for ACTIVE-SF and 6.35 ± 0.18 for ACTIVE-BF). The findings imply that the application of active films may have limited the progression of changes leading to an increase in pH, particularly during the early and intermediate stages of storage.

## 4. Conclusions

In the conducted study, it was demonstrated that gelatin–chitosan films enriched with blackberry fruit (ACTIVE-BF) and sage flower (ACTIVE-SF) extracts represent a promising alternative to conventional non-biodegradable packaging materials used in the food industry. Their development made it possible to combine the structural functions of biopolymers (gelatin and chitosan) with the bioactive properties of natural plant extracts, thereby providing both protective and functional effects.

UV–Vis and FTIR spectroscopy confirmed the effective integration of phenolic compounds into the polymer matrix, indicating that hydrogen bonds were formed between the –OH and –NH groups of the biopolymers and the polyphenols. This phenomenon improved the chemical stability and structural homogeneity of the films while also imparting UV-blocking capacity, which may help protect packaged products against photooxidation.

In terms of physicochemical properties, the developed films exhibited adequate flexibility, relatively low solubility and stable water vapour transmission rate (WVTR), confirming their technological potential. Adding plant extracts did not impair the mechanical properties to an extent that would limit practical application. Moreover, considering the ACTIVE-BF film, an increase in elongation at break was observed, indicating improved flexibility of the material.

The biological activity of the developed films was closely connected with the type of incorporated extract. Both films showed high antioxidant activity, as exhibited by FRAP, DPPH and metal-chelating assays; however, a stronger effect was observed for ACTIVE-SF, which may have been caused by the occurrence of rosmarinic acid and carnosol. Although no unequivocal antimicrobial activity against model microorganisms was detected in the in vitro assays, the in vivo tests performed on salmon fillet samples revealed a real slowdown in the growth of spoilage microflora, particularly yeasts and moulds.

During the nine-day storage of salmon fillets at 4 °C, the active films effectively limited microbial growth and influenced oxidative changes, as reflected by microbiological results, TBARS values and pH stability compared to the control samples. As a result, an extension of microbiological and oxidative shelf-life by approximately two to three days was achieved, relative to traditional LDPE packaging. This indicates that the activity of the films did not result from a classical bactericidal mechanism, but rather from the synergy of antioxidant properties, reduced availability of water and oxygen and local changes in redox conditions on the product surface.

Toxicity and respiratory activity tests confirmed the environmental safety of the developed films, further supporting their application potential in active and biodegradable food-packaging systems.

In summary, the developed films combine protective, active and environmentally friendly functions, offering an alternative to synthetic materials for the packaging of products prone to oxidation, such as fish, meat or vegetable oils. The incorporation of blackberry and sage extracts provides additional functional value resulting from their antioxidant and photoprotective properties.

## Figures and Tables

**Table 1:** Water-related properties of the films.

Parametr	Type of Film		
	BC	ACTIVE-BF	ACTIVE-SF
Thickness [mm]	0.1 ^a^ ± 0.0	0.2 ^a^ ± 0.0	0.2 ^a^ ± 0.0
Water content [%]	13 ^a^ ± 2	14 ^a^ ± 2	13 ^a^ ± 1
Solubility [%]	41 ^a^ ± 5	48 ^a^ ± 2	48 ^a^ ± 2
WVTR [g/m^2^ × d]	547 ^a^ ± 16	531 ^a^ ± 17	545 ^a^ ± 21

Data are presented as mean ± SD; a—distinct letters within individual rows denote differences in statistical significance between means (*p* ≤ 0.05).

**Table 2:** Mechanical properties of the films.

Parameter	Type of Film		
	BC	ACTIVE-BF	ACTIVE-SF
Max breaking load [N]	67.7 ^a^ ± 4.2	63.2 ^a^ ± 7.6	60.2 ^a^ ± 6.3
Tensile strength [kN/m]	4.5 ^a^ ± 0.3	4.2 ^a^ ± 0.5	4.0 ^a^ ± 0.4
Elongation at break [%]	6.1 ^a^ ± 0.7	16.7 ^b^ ± 2.9	6.0 ^a^ ± 1.8
Young’s modulus [N/mm^2^]	1016.4 ^b^ ± 59.5	647.0 ^a^ ± 117.0	832.9 ^ab^ ± 120.5

Data are presented as mean ± SD; a,b—distinct letters within individual rows denote differences of statistical significance between means (*p* ≤ 0.05).

**Table 3:** Antioxidant properties of the films.

Parameter	Type of Film		
	BC	ACTIVE-BF	ACTIVE-SF
FRAP [mM equivalents Trolox/mg]	0.6 ^a^ ± 0.1	17.8 ^b^ ± 0.2	40.8 ^c^ ± 3.3
DPPH [%]	12.8 ^a^ ± 1.0	42.6 ^b^ ± 2.7	46.9 ^b^ ± 0.0
Metal chelating activity [%]	0.0 ^a^ ± 0.0	36.0 ^b^ ± 1.9	50.8 ^c^ ± 1.1

Data are presented as mean ± SD; a,b,c—distinct letters within individual rows denote differences of statistical significance between means (*p* ≤ 0.05).

**Table 4:** Equations for respiratory activity within assessment of biodegradation and phytotoxicity of biocomposites.

Parameter	Type of Film		
	BC	ACTIVE-BF	ACTIVE-SF
Respiratory activity			
y (0–48 h)	1.9721x − 1.4192	1.6989x − 3.4577	1.6989x − 3.4577
R^2^ (0–48 h)	0.9197	0.9157	0.9157
y (49–672 h)	0.1295x + 87.758	0.1339x + 78.662	0.1292x + 79.407
R^2^ (49–672 h)	0.993	0.991	0.994
RA_28_ [mg O_2_/g]	177.2 ^a^ ± 12.2	167.4 ^a^ ± 10.6	166.6 ^a^ ± 9.9
Toxixity			
RA_8_ [mg O_2_/100 seeds]	103.5 ^a^ ± 2.5	64.4 ^b^ ± 5.2	59.4 ^b^ ± 4.8
Fractional composition of humic compounds [g·kg^−1^ d.m.]			
C EXTRACT	70.8 ^a^ ± 0.3	74.8 ^b^ ± 1.2	77.3 ^c^ ± 1.5
C HUMIC ACIDS	32.5 ^a^ ± 0.8	37.6 ^b^ ± 1.1	38.8 ^b^ ± 1.6
C FULVIC ACIDS	38.2 ^b^ ± 0.6	37.2 ^a^ ± 0.2	38.4 ^b^ ± 0.2
Ckh/Ckf RATIO	0.9 ^a^ ± 0.0	1.0 ^b^ ± 0.0	1.0 ^b^ ± 0.0

Data are presented as mean ± SD; a,b,c—distinct letters within individual rows denote differences in statistical significance between means (*p* ≤ 0.05). RAx—cumulative oxygen consumption recorded during respiratory activity over an x-day period (mg O_2_/g d.m.). R^2^—coefficient of determination. y = ax + b, where x—time [h]; [mg O_2_/g d.m.].

**Table 5:** Variations in pH and TBARS of salmon fillets wrapped in edible films.

Sample	Day	TBARS (mg/kg)	pH
0	0	0.3 ± 0.0	6.3 ± 0.1
BC		0.3 ^a^ ± 0.1	6.5 ^ab^ ± 0.0
ACTIVE SF	3	0.3 ^a^ ± 0.0	6.4 ^b^ ± 0.1
ACTIVE BF		0.5 ^b^ ± 0.2	6.3 ^a^ ± 0.0
LDPE		0.2 ^a^ ± 0.0	6.5 ^b^ ± 0.1
BC		0.3 ^a^ ± 0.0	6.6 ^ab^ ± 0.2
ACTIVE SF	6	0.4 ^a^ ± 0.1	6.3 ^ac^ ± 0.1
ACTIVE BF		0.6 ^b^ ± 0.2	6.2 ^a^ ± 0.0
LDPE		0.2 ^c^ ± 0.0	6.5 ^bc^ ± 0.0
BC		0.5 ^b^ ± 0.6	6.2 ^a^ ± 0.0
ACTIVE SF	9	1.4 ^d^ ± 0.4	6.4 ^a^ ± 0.2
ACTIVE BF		0.8 ^c^ ± 0.3	6.4 ^a^ ± 0.2
LDPE		0.3 ^a^ ± 0.0	6.7 ^a^ ± 0.1

Lowercase letters (a,b,c,d) indicate differences between groups on same day. BC—salmon flakes, edible film coating, no extract; ACTIVE SF—salmon flakes, edible film coating, SF extract addition; ACTIVE BF—salmon flakes, edible film coating, BF extract addition; LDPE—salmon flakes, LDPE coating.

## Data Availability

The original contributions presented in this study are included in the article. Further inquiries can be directed to the corresponding author.
